# Neonatal DSP-4 Treatment Modifies Antinociceptive Effects of the CB_1_ Receptor Agonist Methanandamide in Adult Rats

**DOI:** 10.1007/s12640-012-9323-x

**Published:** 2012-04-10

**Authors:** Eva Korossy-Mruk, Katarzyna Kuter, Przemysław Nowak, Ryszard Szkilnik, Monika Rykaczewska-Czerwinska, Richard M. Kostrzewa, Ryszard Brus

**Affiliations:** 1 Chair and Department of Pharmacology, Medical University of Silesia, H. Jordana 38, 41-808 Zabrze, Poland; 2 Department of Neuro-Psychopharmacology, Institute of Pharmacology, Polish Academy of Sciences, Smętna 12, 31-343 Kraków, Poland; 3 Occupational Health Protection Unit, Public Health Faculty, Medical University of Silesia, Medyków 18, 40-752 Katowice Ligota, Poland; 4 Department of Basic Medical Sciences, Medical University of Silesia, Piekarska 18, 41-902 Bytom, Poland; 5 Department of Pharmacology, Quillen College of Medicine, East Tennessee State University, P.O. Box 70577, Johnson City, TN 37614 USA; 6 Department of Nurse, High School of Strategic Planning, Kościelna 6, 41-303 Dąbrowa Górnicza, Poland

**Keywords:** Noradrenergic, Lesion, CB_1_ receptor, Antinociception, Rats

## Abstract

To study the influence of the central noradrenergic system on antinociceptive effects mediated by the CB_1_-receptor agonist methanandamide, intact rats were contrasted with rats in which noradrenergic nerves were largely destroyed shortly after birth with the neurotoxin DSP-4 [*N*-(-2-chloroethyl)-*N*-ethyl-2-bromobenzylamine (50 mg/kg sc × 2, P1 and P3); zimelidine (10 mg/kg sc, 30 min pretreatment, selective serotonin reuptake inhibitor). When rats attained 10 weeks of age, monoamine and their metabolite concentrations were determined in the frontal cortex, thalamus, and spinal cord by an HPLC/ED method. Antinociceptive effects after methanandamide (10 mg/kg ip) apply were evaluated by a battery of tests. In addition, immunohistochemistry and densitometric analysis of the cannabinoid CB_1_ receptor in the rat brain was performed. DSP-4 lesioning was associated with a reduction in norepinephrine content of the frontal cortex (>90 %) and spinal cord (>80 %) with no changes in the thalamus. Neonatal DSP-4 treatment produced a significant reduction in the antinociceptive effect of methanandamide in the tail-immersion test, hot-plate test and writhing tests. In the paw pressure and formalin hind paw tests results were ambiguous. These findings indicate that the noradrenergic system exerts a prominent influence on analgesia acting via the cannabinoid system in brain, without directly altering CB_1_ receptor density in the brain.

## Introduction

Cannabinoids exert palliative effects in cancer patients by stimulating appetite and by abating nausea, vomiting, and pain. To date, cannabinoids have been licensed for clinical use as palliative treatment of chemotherapy, but increased evidence indicates a direct antiproliferative action of these drugs on several tumor cell lines, both in vitro and in animal models (Walsh et al. 2003). Also, recent evidence suggests that the endocannabinoid system mediates stress responses and regulates emotional homeostasis, in part, by targeting noradrenergic circuits. Simultaneously, the midbrain locus coeruleus (LC), that contains most noradrenergic neurons and projects to multiple cortical, limbic and autonomic-related brain structures, regulates arousal, attention, vigilance, stress, and pain (Berridge and Waterhouse 2003; Dunn et al. 2004). Alteration of norepinephrine (NE) exocytosis in the thalamus, brain stem and other nuclei alters output of nociceptive information to higher brain centers from projection neurons. Also, LC stimulation, which increases NE release in spinal cord, inhibits nociceptive transmission in the dorsal horn via α_2_-adrenergic receptors (Cenci et al. 1992; Delaney et al. 2007). Clinical evidence indicates a link between NE with pain modulation and opioid withdrawal syndrome. Abrupt cessation of opioid intake precipitates opioid withdrawal, which produces several aversive responses and symptoms, i.e., an abnormal increase in pain sensitivity (hyperalgesia) (Van Bockstaele et al. 2008).

LC activity is tightly controlled by presynaptic α_2_ autoreceptors (Lichtman and Martin 1991; Pudovkina and Westerink 2005) and by afferent pathways from several brain areas (Lee et al. 2005). Among these, serotonin (5-HT) and γ-aminobutyric acid (GABA)-containing neurons appear to play a major role (Aston-Jones et al. 1991). LC neurons possess a high density of postsynaptic mu-opioid receptors (Van Bockstaele and Commons 2001); and cannabinoids modulate noradrenergic neuronal activity. Scavone et al. (2010) provided evidence for a heterogeneous distribution of CB_1_ receptors in the LC and demonstrated that this receptor and mu-opioid receptors co-exist in cellular profiles in this region. Others have shown an interaction between the cannabinoid system and the NE system in areas such as the prefrontal cortex (Oropeza et al. 2007), nucleus accumbens (Carvalho et al. 2010) and the nucleus of the solitary tract (Jelsing et al. 2009).

Previously, we showed that *N*-(-2-chloroethyl)-*N*-ethyl-2-bromobenzylamine (DSP-4; 50 mg/kg sc per day), administered on the 1st and 3rd days of postnatal life, alters noradrenergic input to the hippocampus and prefrontal cortex (endogenous NE content was reduced by 98.5 and 95.0 %, respectively), without impairing dopaminergic and serotoninergic input to these regions. An elevated NE level was found in brainstem and cerebellum of these DSP-4-treated rats, suggestive of reactive neuronal sprouting (Brus et al. 2004; Nowak et al. 2006; Dąbrowska et al. 2007). In addition, we reported that neonatal DSP-4 treatment modifies convulsant effects of bicuculine and pentetrazole, as well as sensitivity to the anxiolytic-like effect of benzodiazepines, and the sedative-hypnotic effect of phenobarbital and ethanol in adult rats (Bortel et al. 2007, 2008a, b). And we established that the GABA transaminase inhibitor vigabatrin causes a twofold increase in the extracellular GABA concentration in DSP-4-lesioned rat brain (Bortel et al. 2008c). In the present study, a lesion of noradrenergic neurons was made in early postnatal ontogeny, in order to assess how subsequent postnatal compensatory processes influence cannabinoid-induced antinociception. This phenomenon may be of importance, because adrenergic receptor dysfunction is suspect in patients with clinical depression, anxiety disorder and other neuropsychiatric disorders (Ressler and Nemeroff 1999; Anand and Charney 2000) and neurological disease (Parkinson’s and Alzheimer’s disease) (Narabayashi 1999; Haglund et al. 2006). Furthermore, the difficulties of relieving painful symptoms associated with some pathological entities, i.e., bone cancer pain, justify experimental efforts to define new analgesic targets. Cannabinoid CB_1_ receptor agonists represent a class with this desired analgesic potential (Farquhar-Smith 2009).

## Materials and Methods

### Animals and Treatment

Wistar rats (University Animal Department, Katowice, Poland) were housed under controlled environmental conditions, in a well-ventilated room, at 22 ± 2 °C and under a 12 h light:12 h dark cycle (lights on from 7:00 a.m. to 7:00 p.m.). Animals received food and water ad libitum. Litters remained with dams until the 21st day after birth and then were placed in individual cages according to sex. Experiments were carried out in the morning in only male rats, handled in accordance with the principles and guidelines described in the *NIH* *Guide for the Care and Use of Laboratory Animals*. All procedures were reviewed and approved by the Local Bioethical Committee for Animal Care at the Medical University of Silesia (decision no. 49/2009 issued on 17.06.2009).

The central noradrenergic system of newborn rats was lesioned with DSP-4 (Sigma, St. Louis, MO, USA). Rats were injected on the 1st and 3rd day of postnatal life with either DSP-4 (50 mg/kg sc) or 0.9 % NaCl (1.0 ml/kg sc). DSP-4 was dissolved in distilled water immediately before injection, and preceded 30 min beforehand by treatment with the selective serotonin reuptake inhibitor zimelidine (10 mg/kg ip) (Sigma, St. Louis, MO, USA)—in order to prevent serotoninergic effects of DSP-4. The dose and the days of treatment were selected on the basis of the reports by Jonsson et al. (1982), Brus et al. (2004) and Dąbrowska et al. (2007). Rats continued to be housed as above until 10 weeks, for further experimentation.

### Assessment of Biogenic Amine and Metabolite Content

At 10 weeks after birth control and DSP-4-treated rats were injected with saline (1.0 ml/kg ip) or methanandamide (10 mg/kg ip) (Tocris Bioscience, Ellisville, MO, USA), and were terminated by decapitation 60 min later. The frontal cortex, thalamus, and spinal cord were rapidly dissected and placed on dry ice, weighed and stored at −70 °C, pending assay. Samples were homogenized for 15–20 s in ice-cold trichloracetic acid (0.1 M), containing 0.05 mM ascorbic acid. After centrifugation (5,000×*g*, 5 min), supernatants were filtered through 0.2 μm cellulose membranes (Titan MSF Microspin filters, Scientific Resources Inc., Eatontown GB) and filtrates were injected onto the HPLC/ED column. Levels of NE, dopamine (DA), 3,4-dihydroxyphenylacetic acid (DOPAC), 5-hydroxytryptamine (5-HT) and 5-hydroxyindoleacetic acid (5-HIAA) were assayed by HPLC/ED (Nowak et al. 2003). The composition of the mobile phase was: 75 mM NaH_2_PO_4_, 1.7 mM 1-octanesulfonic acid, 5 μM EDTA (Avocado, Research Chemical Ltd., Morecambe, GB), 100 μl triethylamine (Sigma, St. Louis, USA), 9.5 % acetonitrile (J.T. Baker, Deventer, Holland), pH 3 adjusted with phosphoric acid (Fluka, Steinheim, Switzerland). The flow rate was maintained at 0.7 ml/min, at a temperature of 22 °C, and the oxidation potential was fixed at +700 mV, 10 nA/V sensitivity. Peaks were automatically integrated by universal chromatographic interface UCI-100 (Dionex Softron Gmbh, Germering, Germany). The instrumentation included an electrochemical detector (Gilson, Villiers-le-Bel, France) model 141 with flow cell, piston pump model 302 with head 5SC (Gilson, Villiers-le-Bel, France), manometric module model 802 (Gilson, Villiers-le-Bel, France), thermostat for STH 595 column (Dionex Softron Gmbh, Germering, Germany), precolumn Hypersil BDS C18, 10 × 4 mm, 3 μm (ThermoQuest, Waltham, GB), and chromatographic column Hypersil BDS C18, 250 × 4.6 mm, 3 μm (ThermoQuest, Waltham, GB). The data were quantified using the area under the peaks and external standards, using Chromeleon software (Dionex, Germany).

### Immunohistochemistry and Densitometric Analysis

Immunostaining for CB_1_ receptor expression was carried out according to Kuter et al. (2011). At 10 weeks after birth control and DSP-4-treated rats were decapitated. The brains were rapidly removed, postfixed in cold 4 % paraformaldehyde for 7 days and cryoprotected in 20 % sucrose solution in phosphate-buffered saline (PBS). The brains were cut on a freezing microtome into 30 μm frontal sections (AP = 2.52 mm from bregma according to Paxinos and Watson 2007). Free-floating sections were incubated for 48 h at 4 °C in a primary antibodies (anti-CB_1_ receptor, 1: 1,000; Sigma, Germany), rinsed in PBS, then incubated for 30 min in secondary antibodies (anti rabbit biotinylated, 1:200, Vector Laboratories, UK) and processed using an ABC-peroxidase kit (Vector Laboratories, UK) and 3,3′-diaminobenzidine as a chromogen. The stained sections were mounted on slides, dried, dehydrated, cleared in xylene and cover-slipped in a Permount medium (Fisher Scientific, USA).

Two sections stained immunohistochemically for CB_1_ receptor were analyzed densitometrically in the region of striatum and frontal cortex. The sections were scanned all together, digitalized, adjusted for brightness, and regions of interest outlined using Multi Gauge programme (FUJIFILM). The optical density and area (OD) were counted. Background signal was subtracted from each section separately, from the region of corpus callosum. The results are presented as mean of each OD/area^2^ value minus backgrounds.

### Tail-Immersion Test

Antinociception was evaluated by measuring response latencies in the warm water tail-immersion (tail-flick) assay (Janssen et al. 1963). Each animal was placed in a cone restrainer, and the caudal 2/3 of tails of rat was immersed 5 cm in a 56 °C water bath. The time for the rats to remove their tails from the water was expressed as the tail flick latency. A cut-off time of 10 s was used to minimize damage to the skin of the tail. Reaction latency (s) was used as a parameter reflecting the intensity of the pain experienced. The determined latency time for each animal was converted to the percentage of analgesia according to the formula:

$$ {\text{\% analgesia = }}\frac{{T_{\text{x}} - \, T_{0} }}{{T_{ \max } - \, T_{0} }} \times 100 $$

*T*_x_—the individual latency time determined at appropriate intervals after examined analgesics administration, *T*_0_—individual latency time determined before analgesics injection, *T*_max_—10 s. The analgesic effect was measured before drug administration (after saline 1.0 ml/kg ip) and at 30, 60, 90, 120, and 150 min after methanandamide (10 mg/kg ip) injection.

### Hot Plate

Antinociception was assessed according to O’Callaghan and Holtzman (1975) using a hot-plate instrument (COTM, Bialystok, Poland) with the plate temperature maintained at 56 ± 0.1 °C. The rat was placed with all four paws on the plate, and the latency time to licking or shaking a hind limb was measured. The cut-off time was set at 20 s to avoid tissue damage. The determined latency time for each animal was converted to the percentage of analgesia according to the formula:

$$ \% {\text{ analgesia }} = \frac{{T_{\text{x}} - \, T_{0} }}{{T_{ \max } - \, T_{0} }} \times 100 $$

*T*_x_—the individual latency time determined at appropriate intervals after examined analgesics administration, *T*_0_—individual latency time determined before analgesics injection, *T*_max_—20 s. The analgesic effect was measured before drug administration (after saline 1.0 ml/kg ip) and at 10, 20, 30, 40, 50 and 60 min after methanandamide (10 mg/kg ip) injection.

### Writhing Test

Control and DSP-4 rats (deprived of food 24 h before testing) were placed individually in clear plexiglas boxes (40 × 30 × 20 cm) and allowed to acclimate for 30 min. Rats were then injected with saline (1.0 ml/100 g ip) and 30 min later, treated with ethacrynic acid solution (3.0 mg/1 ml/100 g) in the left lower quadrant of the abdomen. Ethacrynic solution was prepared ex tempore in the proportion of 3/47 ethanol/water. Rat were returned to the chamber and 10 min later contractions of abdominal musculature (writhes) were counted (contractions of the abdomen, twisting and turning of the trunk, arching of the back and extension of the hind limbs) for the following 60 min with division on 10 min intervals (10–20, 20–30, 30–40, etc.). Rats were used once and then killed immediately (Korzeniewska-Rybicka and Płaźnik 2001). According to the same paradigm, separate groups of rats (control and DSP-4) were tested after methanandamide (10 mg/kg ip) treatment. The degree of antinociception was expressed as the percentage decrease in the number of writhes and was calculated according to the formula:

$$ {\text{\% inhibition of writhing = 100}} - \frac{100 \times B}{A} $$

*A*—the mean number of writhes in saline-treated control and DSP-4 rats for appropriate observation period; *B*—the mean number of writhes in drug-treated rats counted for appropriate observation interval.

### Paw-Pressure Test

Nociceptive thresholds in rats were determined by a modification of the Randall–Selitto method (Randall and Selitto 1985), the paw-pressure vocalization test, in which a constantly increasing pressure is applied to the hind paw until the rat squeaks. The Ugo Basil analgesimeter (probe tip diameter 1 mm; weight 25 g) was used. A 750-g cutoff value was used for preventing tissue damage. In brief, a constantly increasing pressure was applied to the right hind paw of the rat at the metacarpal level between the third and the fourth finger to determine the minimum stimulus necessary to evoke an obvious nociceptive response (a sharp paw withdrawal). Rats were habituated to the full procedure on two consecutive days and experiments were conducted on the third day. To insure nociceptive threshold stability, basal nociceptive threshold was measured three times (with an interval of 30 min) on the 2 days before the planned experimental day. On the experimental day, basal nociceptive threshold was also determined three times before drug injections. The following formula was used to count the percentage of analgesia:

$$ \% {\text{ analgesia }} = \frac{100 \times B}{A} - 100 $$

*A*—mean pressure (g) from 3 assessments before drug administration; *B*—pressure (g) assessed at 30, 60, 90, 120 min after methanandamide (10 mg/kg ip) treatment.

### Formalin Test

Inflammatory pain and analgesia were determined using the formalin test in the rat (Acton et al. 1992). Rats were placed in a clear plastic chamber (30 × 30 × 30 cm) for 30 min to allow them to accommodate to their surroundings with a mirror placed at a 45° angle beneath the floor to allow an unobstructed view of the paws. Then, 30 min after methanandamide (10 mg/kg ip) administration, 50 μl of 5 % formalin solution was injected subcutaneously, 30-gauge needle, into the right hind paw plantar surface. Rats were then returned to the chambers, and nociceptive behavior was observed immediately after formalin injection. Nociceptive behavior was quantified using the scale 0–3 points. Formalin-induced pain is biphasic. The initial acute phase (0–10 min) is followed by a relatively short quiescent period, which is then followed by a prolonged tonic response (15–60 min). A reduction of formalin-induced behavior observed after administration of a given drug is interpreted as an analgesic response.

### Statistical Analysis

Group differences in monoamines were assessed by an analysis of variance (ANOVA) and the post-ANOVA test of Newman–Keuls. Group differences in behavioral studies were analyzed by Student’s *t* test. A *P* value <0.05 was taken as the level of significant difference.

## Results

### Effect of DSP-4 Treatment on Monoamine Concentration in the Frontal Cortex, Thalamus, and Spinal Cord

**Fig. 1:**
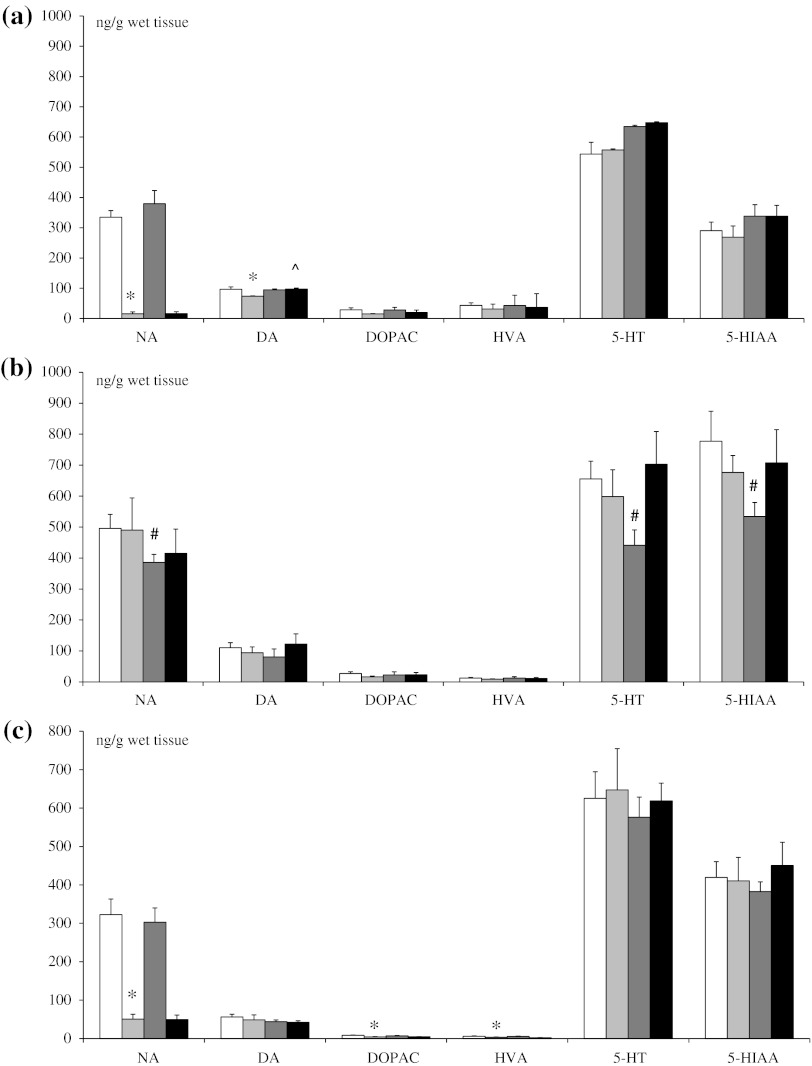
Effect of neonatal DSP-4 lesioning (50 mg/kg sc on the 1st and 3rd days of postnatal life) on the content of monoamines and their metabolites in frontal cortex (**a**), thalamus (**b**) and spinal cord (**c**), following acute methanandamide (10 mg/kg ip) treatment of adult rats (*x* ± SEM; *n* = 6). Legend *White square* control, *Light grey square* DSP-4 control + methanandamide, *Dark grey square* DSP-4 + methanandamide, *P < 0.05, control versus DSP-4, ^#^ *P* < 0.05, control + methanandamide versus DSP-4 + methanandamide, ^*P* < 0.05, DSP-4 versus DSP-4 + methanandamide

In rats treated on the 1st and 3rd days of postnatal life with DSP-4 (50 mg/kg sc), and killed at 10 weeks, the adulthood level of NE in the frontal cortex and spinal cord either after saline or methanandamide (10 mg/kg ip) treatment was significantly decreased in comparison to the respective control. DA content in the frontal cortex was reduced in the DSP-4 group while DOPAC and HVA were reduced in spinal cord, versus control. The endogenous level of 5-HT and 5-HIAA in both structures remained unaltered (Fig. 1a, c).

In the thalamus methanandamide treatment significantly reduced NE, 5-HT and 5-HIAA concentration only in control rats, while there was no consisted change in DA and its metabolite DOPAC (Fig. 1b).

### Immunohistochemistry and Densitometric Analysis for CB_1_ Receptor Expression

**Fig. 2:**
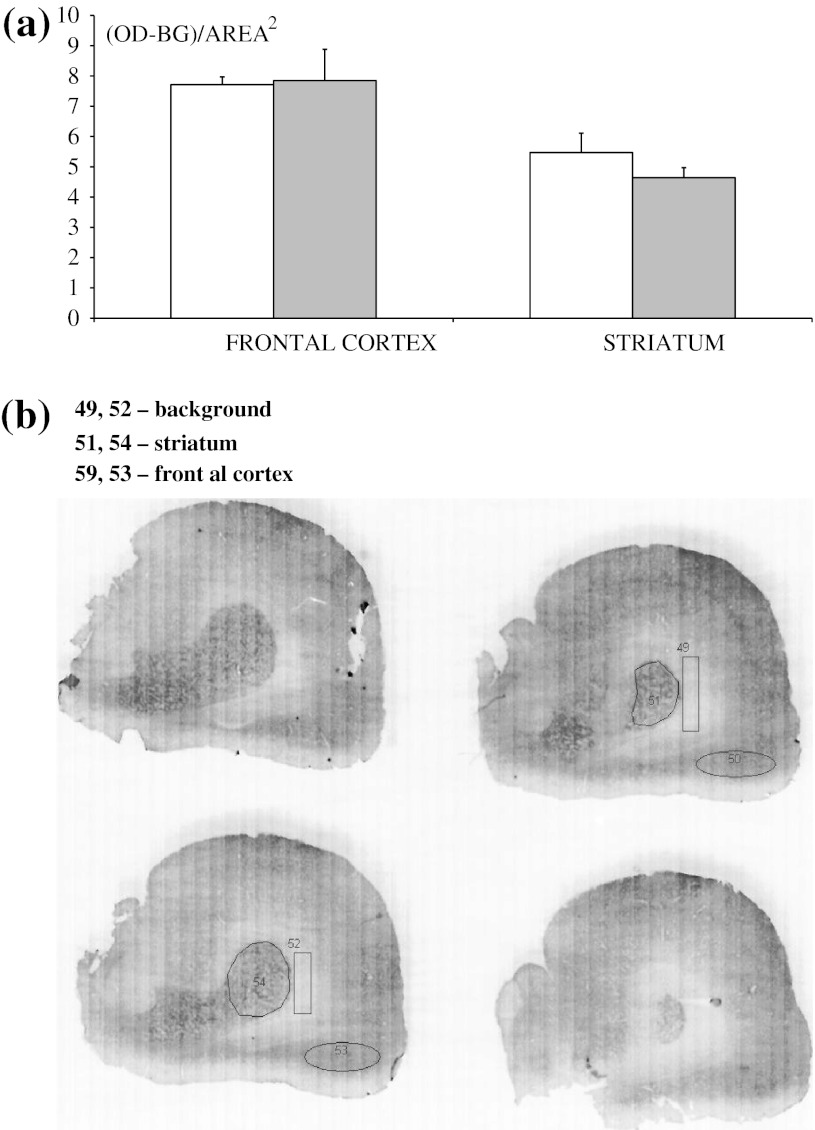
Effect of neonatal DSP-4 lesioning (50 mg/kg sc on the 1st and 3rd days of postnatal life) on CB_1_ receptor immunoreactivity, assessed densitometrically in brain slices in the frontal cortex and striatum of adult rats. **a** Results are shown as the mean ± SEM (*n* = 7 per group) in arbitrary units of optical density (OD). **b** Representative CB_1_ receptor immunostained sections at the level of the corpus striatum with delineation of subregions of the corpus striatum and frontal cortex. Background was assessed in the region (shown as a *rectangle*) of the corpus callosum. Legend *White square* control, *Grey square* DSP-4

Immunostaining for CB_1_ receptor expression was carried out in the frontal cortex and striatum, showing no significant changes between control and DSP-4 rats in both structures (Fig. 2).

### Tail-Immersion Test

**Fig. 3:**
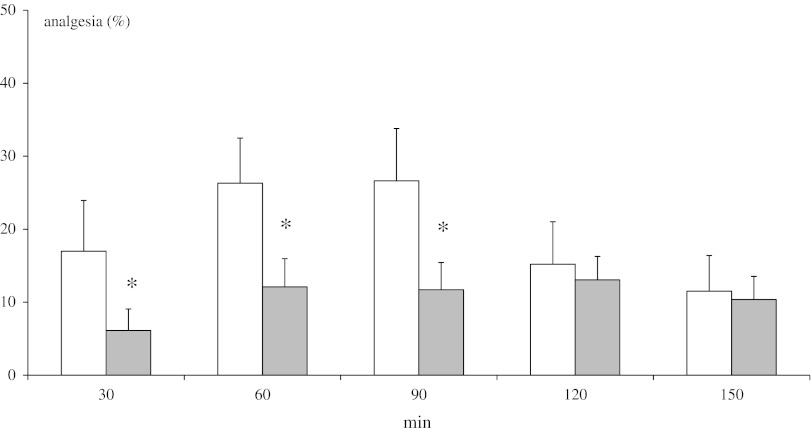
Effect of neonatal DSP-4 lesioning (50 mg/kg sc on the 1st and 3rd days of postnatal life) on antinociception effects assessed in the tail-immersion test after methanandamide (10 mg/kg ip) treatment in rats (mean ± SEM; *n* = 8). Legend *White square* control, *Grey square* DSP-4, *P < 0.05, control versus DSP-4

Methanandamide (10 mg/kg ip) evoked a lower antinociceptive effect in DSP-4 rats in comparison to control rats. Obtained differences were significant at 30, 60, and 90 min of testing (Fig. 3).

### Hot-Plate Test

**Fig. 4:**
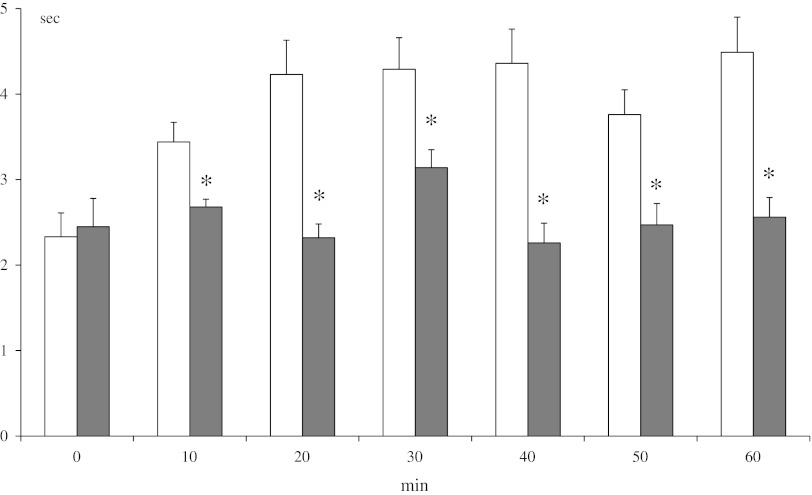
Effect of neonatal DSP-4 lesioning (50 mg/kg sc on the 1st and 3rd days of postnatal life) on antinociception effects assessed in the hot-plate test after methanandamide (10 mg/kg ip) treatment in rats (mean ± SEM; *n* = 8). Legend as in Fig. 3

Methanandamide (10 mg/kg ip) elicited a lower antinociceptive effect in DSP-4 rats in comparison to control animals, and differences were significant at 10, 20, 30, 40, 50 an 60 min of observation (Fig. 4).

### Writhing Test

**Fig. 5:**
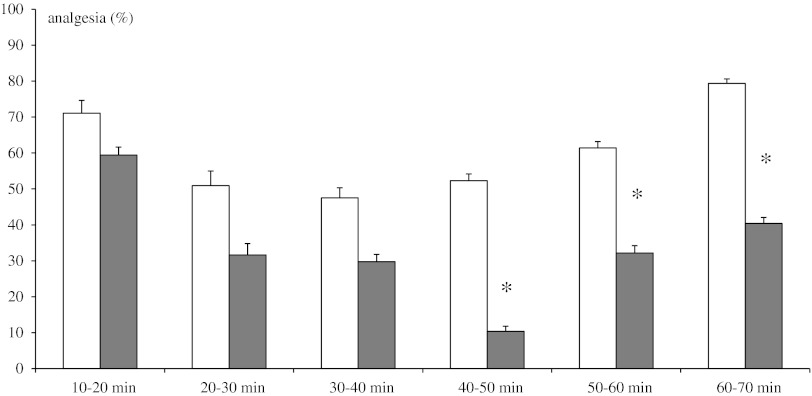
Effect of neonatal DSP-4 lesioning (50 mg/kg sc on the 1st and 3rd days of postnatal life) on antinociception effects assessed in the writhing test after methanandamide (10 mg/kg ip) treatment in rats (mean ± SEM; *n* = 8). Legend as in Fig. 3

Injections of methanandamide (10 mg/kg ip) produced a lower antinociceptive effect in the DSP-4 group in comparison to control rats, and the effect was significant at 40–50, 50–60 and 60–70 intervals of observation (Fig. 5).

### Paw-Pressure Test

**Fig. 6:**
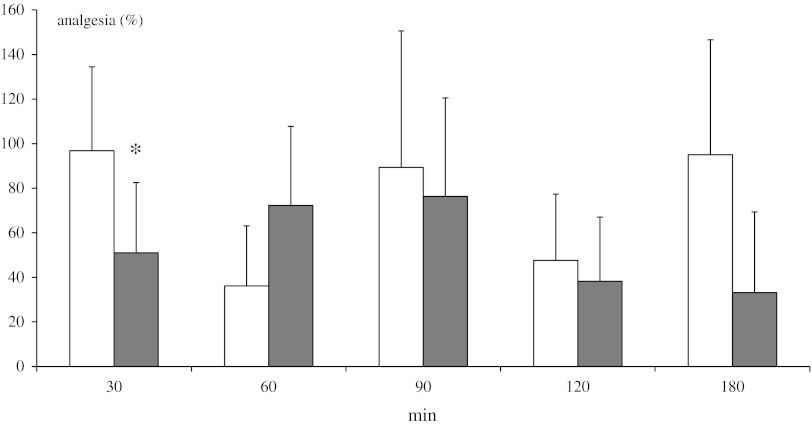
Effect of neonatal DSP-4 lesioning (50 mg/kg sc on the 1st and 3rd days of postnatal life) on antinociception effects assessed in the paw-pressure test after methanandamide (10 mg/kg ip) treatment in rats (mean ± SEM; *n* = 8). Legend as in Fig. 3

Prior to drug injection, withdrawal thresholds of intact and DSP-4 rats were 8.94 ± 1.36 and 9.03 ± 1.29 g, respectively (means from all measurements, *n* = 24 for each group). Methanandamide (10 mg/kg ip) elicited a narrowly lower antinociceptive effect in DSP-4 treated rats in comparison to control animals (significant difference only at 30 min of testing) (Fig. 6).

### Formalin Test

**Fig. 7:**
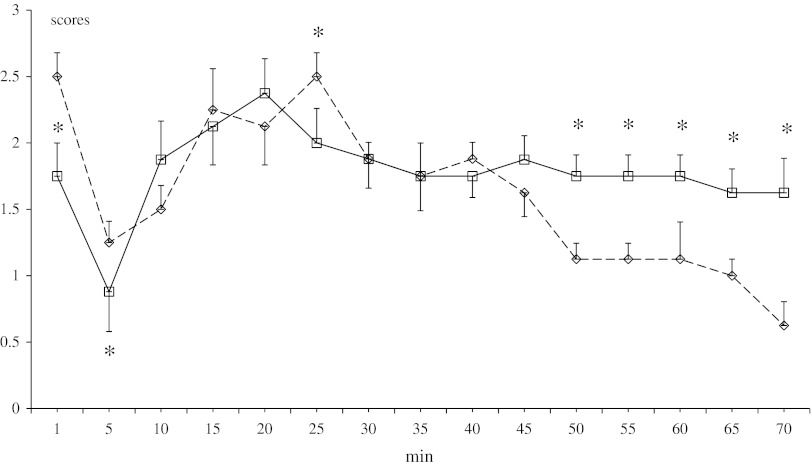
Effect of neonatal DSP-4 lesioning (50 mg/kg sc on the 1st and 3rd days of postnatal life) on antinociception effects assessed in the formalin test after methanandamide (10 mg/kg ip) treatment in rats (mean ± SEM; *n* = 8). Legend as in Fig. 3

To assess the effect of a neonatal noradrenergic lesion on adulthood analgesic action of methanandamide, a comparison of DSP-4 and control rats was made for the behavioral responses to sc injection of 50 μl (5 %) of formalin into a hind paw. Methanandamide (10 mg/kg ip) was administered 30 min before formalin application. Both groups showed the typical biphasic nociceptive response for the 60 min of testing, but DSP-4 lesioned rats scored fewer points (spending less time licking/biting the injected hind paw) in the first phase of the formalin test than the control group (*P* < 0.05 at 1 and 5 min), but in the remaining 50 min of observation the opposite effect was observed (Fig. 7).

## Discussion

The major finding of the present study is that neonatal DSP-4 treatment—resulting in permanent destruction of noradrenergic inputs to spinal cord and cortical areas—diminishes adulthood CB_1_-receptor agonist mediated antinoception, in the absence of change in CB_1_ receptor density in brain.

The antinociceptive effects of endocannabinoids have been well described in animal models of acute and chronic pain (Walker and Huang 2002). Cannabinoid analgesia involves effects at the supraspinal, spinal and peripheral levels (Agarwal et al. 2007; Hohmann and Suplita 2006). Furthermore, there is a large body of evidence associating the cannabinoid system with regulation of noradrenergic activity. Systemic administration of the synthetic cannabinoid agonist WIN 55,212-2 enhances NE release in prefrontal cortex and increases c-fos expression in the LC. In addition, there is a significant alteration in adrenergic receptor and NE transporter expression in the frontal cortex (Oropeza et al. 2005). McLaughlin et al. (2009) found that the acute administration of exogenous cannabinoid ligands activates the hypothalamic–pituitary–adrenal axis through an increase in serotoninergic and noradrenergic neurotransmission. All of the above may underlie the reported changes in attention, cognition, anxiety and pain threshold commonly observed after cannabinoid exposure (Reyes et al. 2009). Yet, there are no data regarding such an association in NE-denervated animals. Neonatal treatment with neurotoxins, such as 6-hydroxydopamine (6-OHDA) or DSP-4 produces marked impairment in development of the central noradrenergic system, i.e., permanent and robust NE-denervation of the frontal cortex and spinal cord (Fig. 1a, c), accompanied by NE hyperinnervation of brainstem and cerebellum (Jaim-Etcheverry and Zieher 1980; Medina and Novas 1983).

In the present study, destruction of noradrenergic neurons by DSP-4 significantly decreased the antinociceptive effect of methanandamide (10 mg/kg ip) in the tail-immersion test, hot-plate test, and writhing test (Figs. 3, 4, 5). Ambiguous results were obtained in the paw pressure and formalin tests (Figs. 6, 7). Despite marked antinociceptive effects of methanandamide, there was no change in CB_1_ receptor density in rat brain (Fig. 2). Gutierrez et al. (2003) demonstrated that intrathecal administration of 6-OHDA, resulting in selective 85 % NE depletion in rat lumbar spinal cord, attenuated the antinociceptive effect of the cannabinoid agonist WIN55,212-2 (5 or 10 mg/kg, ip) as assessed in the tail-flick and formalin tests. They also found that WIN55,212-2 suppressed formalin-evoked fos protein expression, a marker of neuronal activity, in the lumbar dorsal horn of sham-operated rats, while no suppression was observed in lesioned rats. Thus, cannabinoids produce antinociception, in part, by modulating descending noradrenergic systems. To the best of our knowledge there are no data relating to the analgesic effect of CB_1_ receptor agonists in neonatally DSP-4 treated rats. Kushikata et al. (2011) showed that DSP-4 (50 mg/kg ip in adult rats) significantly reduced ketamine analgesia in the hot-plate test. Others (Zhong et al. 1985) found that in rats pretreated intrathecally with DSP-4, the analgesic effect of morphine, given either icv or ip and assessed in a tail-flick test, was significantly attenuated.

Previous reports (Oropeza et al. 2005; Page et al. 2008) indicate that CB_1_ receptor agonists activate the noradrenergic pathway. Martin et al. (1999) reported that WIN55,212-2 elevated tail-flick latencies when injected into the noradrenergic A5 region. Yoon and Choi (2003) demonstrated synergistic interaction after intrathecal delivery of WIN 55,212-2 and clonidine in the formalin test. And, Tham et al. (2005) showed that an α_2_-adrenoceptor agonist (dexmedetomidine) when combined with a cannabinoid receptor agonist (CP55,940) resulted in a synergistic antinociceptive effect in the hot-plate test. Summing up, the above reports, plus results of the current study, indicate that a reduction in methanandamide induced analgesia may be attributable to destruction of noradrenergic neurons.

Nevertheless, we cannot definitely exclude participation of the 5-HT system in the obtained results, particularly considering the fact that Mendiguren and Pineda (2009) showed that anandamide inhibited 5-HT neuronal firing in rat brain slices of the dorsal raphe nucleus. Also, pain sensation is known to be modified by the serotoninergic system, another monoaminergic pathway. As already noted, DSP-4-induced NE depletion attenuated 5-HT ligand mediated analgesia (Minor et al. 1986; Archer et al. 1987). And in our study we found that in the thalamus methanandamide reduced 5-HT and 5-HIAA concentrations, although only in control rats. Therefore, it is quite possible that the serotoninergic system in particular and other phenotypic systems in general, may be influenced by DSP-4 lesioning of noradrenergic innervation and its effect on antinociception.

In conclusion, the present study demonstrates a prominent effect of noradrenergic neurons in regulating the antinociceptive effects of methanandamide—the CB_1_ receptor agonist—without altering CB_1_ receptor density in brain. The associated pathways involved in this effect remain to be resolved.
